# Supplementary material for: Immunosuppressive niche engineering at the onset of human colorectal cancer
Source: Nat Commun. 2022 Apr 4;13:1798. doi: 10.1038/s41467-022-29027-8 (PMC8979971; doi:10.1038/s41467-022-29027-8)
Supplement: Supplementary file 1 — Supplemental Information [file 41467_2022_29027_MOESM1_ESM.pdf]

Steady state analysis of deterministic mathematical model

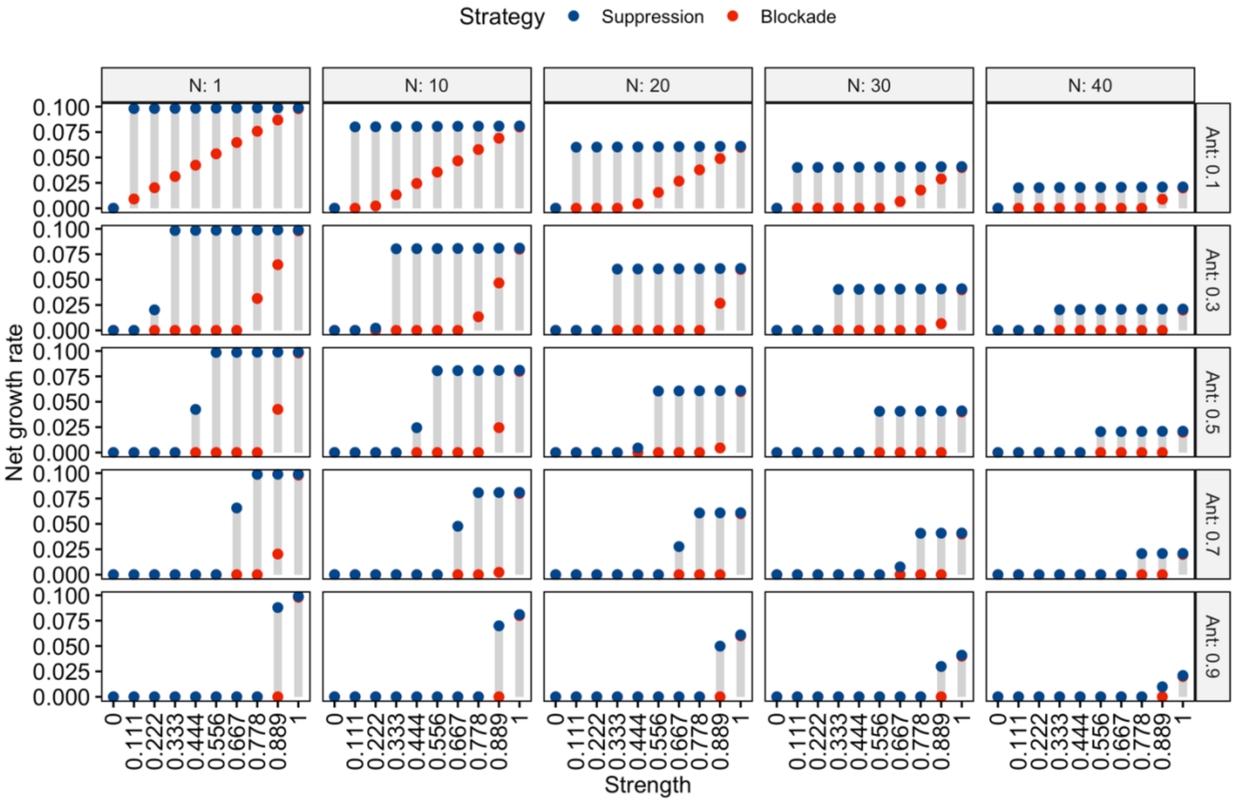

Figure 1. Here, we show the growth rate (i.e. the realized growth rate minus the kill and death rates = $\boldsymbol{r_i}\left(\frac{\boldsymbol{K_i} - \overbrace{\sum_1^j \boldsymbol{\alpha_{ij} N_j}}^{\text{competition}} + \overbrace{\boldsymbol{0.5\sigma_i N_i}}^{\text{growth benefit}}}{\boldsymbol{K_i}}\right) - \boldsymbol{\gamma_i}\overbrace{\left(\boldsymbol{1} - \frac{\sigma_i}{\gamma_i}\right)}^{\text{suppression}}\overbrace{(\boldsymbol{1} - \boldsymbol{\phi_i}\,)}^{\text{blockade}} - \boldsymbol{\delta}$)) for a new cell in a population in a larger population of N cells (columns) that has a carrying capacity K=100. Each cell has an antigenicity (rows) and a different strength of Suppression ($0 \le \sigma \le 1, \phi = 0$) or Blockade ($\sigma = 0, 0 \le \phi \le 1$). The Suppression strategy has a growth advantage over the Blockade strategy, giving it a selective advantage that allows it to outcompete Blockade. Furthermore, Suppression is more capable of invading populations, as it continues to have a positive net growth rate as the overall population approaches K, whereas Blockade must be stronger to grow into larger populations.

In order to determine the long-term outcomes of the deterministic mathematical model we performed a linear steady state of the system in Equation 1. Given we were only interested in the outcome of the competition between two tumor subpopulations at a time (Get Lucky vs Get Smart; Suppression vs Blockade), we simplified the model to its 2-dimensional form, which can be rewritten as a Lotka-Volterra competition model. Subsequently, we used the well-known results about the linear stability of this system to classify the steady states (Gause-Witt isocline analysis (Gause & Witt, 1935)) for details see e.g. (J. D. Murray, 2002). All calculations and plotting were carried out in R.

Stochastic mathematical model

A non-spatial agent-based model (ABM) was developed to simulate tumor evolution under immune predation and escape. Given the large population sizes, a branching process that produced millions of clones wasn't feasible, and so we limited the number of possible phenotypes. Each ith phenotype had 4 distinct traits: antigenicity (γ_i), immune blockade (ϕ_i), immune suppression (σ_i), and number of driver mutations (n_i). Immune blockade/suppression traits were each binary, the maximum number of driver mutations was 4, and there were 10,000 antigenicity values evenly distributed between 0-1. Thus, in total,

there were between and 50,000 - 200,000 unique phenotypes depending on immune suppression and blockade values. For example, if the immune suppression parameter was 0, then there is only one immunosuppression phenotype, but if it is non-zero then there are 2 immunosuppression phenotypes (0 or σ). We assumed that all phenotypes have the same intrinsic death rate, $\delta = 0.01$.

The number of driver mutations, n_i, determined which “species” the phenotype belonged to, which in turn determined the phenotype’s division rate (r_i), carrying capacity (K_i), and intra-species competition (found in the competition matrix, α). If $n_i < 2$, phenotype i belonged to the epithelial species, which had a division rate of $r_i = 0.2$ (Carulli, Samuelson, & Schnell, 2014) and carrying capacity of $K_i = 10^7$; if $2 \leq n_i < 4$, the phenotype was of an adenoma, phenotype i had a division rate of $r_i = 0.2106$ and carrying capacity of $K_i = 10^8$; when $n \geq 4$ phenotype i belonged to the carcinoma species and had division rate $r_i = 0.221$ and carrying capacity $K_i = 10^9$. Increases in division rates were based in differences in observed average KI67 quadrat density between CRA and CRC, which was 1.05. This ratio was applied to the epithelial division rate, and then applied again to get the CRC division rate. The intra-species competition coefficients in α are based on the assumption that epithelial and adenomas occupy different areas, while carcinomas are invasive.

$$\alpha = \begin{array}{cccc} & E & A & C \\ E & 1 & 0 & 1 \\ A & 0 & 1 & 1 \\ C & 1 & 1 & 1 \end{array}$$

Each time step, a phenotype could create mutants, the number of which was determined by drawing from the binomial distribution, where the number of trials was the population size and the probability of success the cellular mutation rate (μ), i.e. $Binomial(N = N_i, p = \mu)$. The cellular mutation rate of was calculated as follows. Exome size was estimated based on information in (M. Lek et al., 2016), which describes the exome as having 45Mb, with 18Mb of possible synonymous variants. Therefore, we modeled the 45Mb-18Mb=27Mb base pairs that may produce non-synonymous variants. The “normal” per division base pair mutation rates were set to $2.91(10^{-9})$ (Monkol Lek et al., 2016; Werner et al., 2020). Given these values, we defined the cell mutation rate to be the probability of at least one non-synonymous mutation in the exome per cell per division: $0.0756 = 1 - Binomial(k = 0, N = 2.7e7, p = 2.91(10^{-9}))$.

We assume all mutations are inherited, and so all mutants have a higher antigenicity that their parent. In addition, mutants may also acquire one of three types of beneficial mutations: driver mutations, the ability to protect from T-cell attack, and the ability to recruit immunosuppressive cells. The multinomial distribution was used to determine how many of each mutant to make. Given that the number of genes in the genome has been estimated to be $g = 20412$ (Cunningham et al., 2019), the probability of creating a cell that mutated an immunosuppressive gene is $\frac{1}{g}$. Likewise, the probability of creating a cell that mutated an immune blockade gene is also $\frac{1}{g}$.If there are 25 possible driver genes, then the probability a mutant has an additional driver mutation is $\frac{25-n_i}{g}$. Finally, the probability that the new cell didn’t mutate any of these beneficial mutations, and only acquired a neoantigen, was $\frac{g-27+n_i}{g}$.

Therefore $\vec{m} = Multinomial\left(N = u_{ij}^t, \vec{\mu}\right)$ is a four element vector where: $\vec{m}_1$ is the number of clones to create that do not have a beneficial mutation; $\vec{m}_2$ is the number of clones to create that acquire a driver mutation, with m_{ij} being the number of driver mutations N_{ij} has accumulated; $\vec{m}_3$ is the number of clones to create that express checkpoint inhibitors; and $\vec{m}_4$ is the number of clones to create that recruit immunosuppressive cells. The parameter n is the number of genes in the genome, and n_{driver} is the number of possible driver mutations.

The vector $\vec{u}$ contains the probability for each mutation type, with

$$\vec{u} = \left[1 - \frac{n_{\text{driver}} - m_{ij} - 2}{n}, \frac{n_{\text{driver}} - m_{ij}}{n}, \frac{1}{n}, \frac{1}{n}\right]$$

Note that calculation of $\vec{m}_2$ assumes the number of driver mutations that will have an effect decreases as driver mutations are accumulated. In other words, it is assumed that if a driver is “hit” more than once, the benefit comes only with the first mutation, and subsequent hits have no effect. It is also assumed there is one gene for protecting from T-cell attack, and one gene for recruiting immunosuppressive cells.

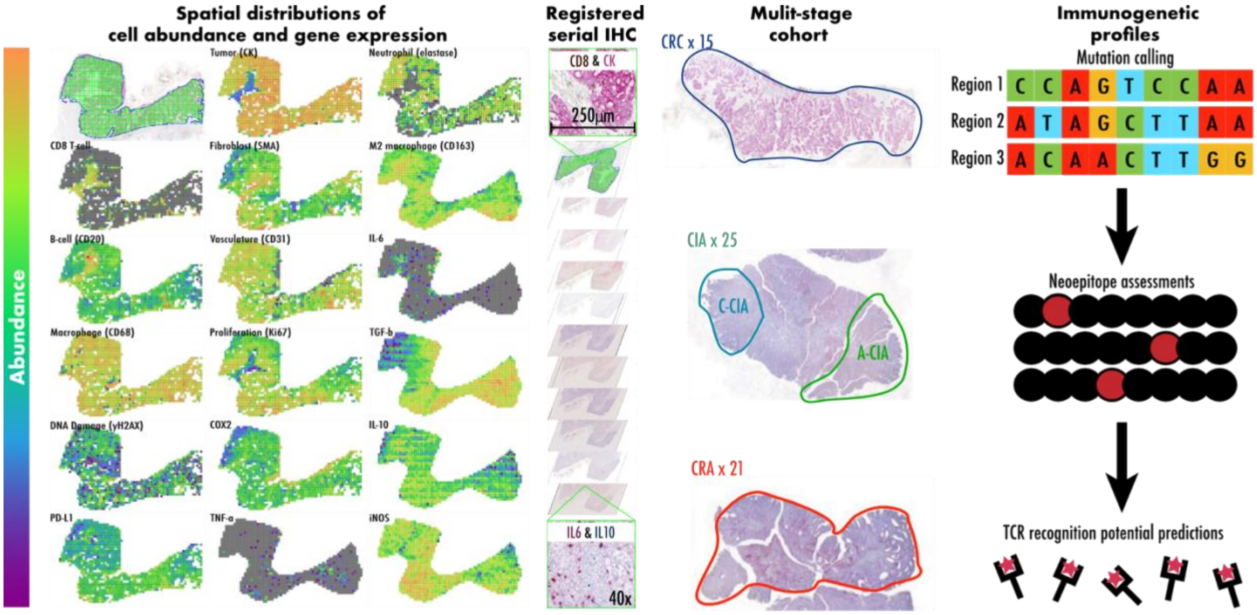

Figure 2. We studied changes in the tumor ecology and antigenic intra-tumor heterogeneity (aITH) using a cohort of 12 colorectal adenomas (CRA), 15 carcinomas (CRC), and 25 intermediate ca-in-ads (CIA), which have a carcinoma (C-CIA) emerging from its (presumably) ancestral adenoma (A-CIA). Given this unique dataset, we were able to determine what distinguishes benign adenomas (CRA) from those that progress (A-CIA), and what changes occur during progression from late adenoma (A-CIA) to nascent carcinoma (C-CIA) to mature carcinoma (CRC). Changes in the ecology were characterized using 17 cell markers and RNA transcripts associated with cytokine expression measured on whole slide images. Spatial analysis was conducted by registering the thin serial slices to create a spatially aligned composite image. The composite image was subsequently divided into 250μm x 250μm quadrats, and abundance estimated as being proportional to the number of pixels positive for each marker at 40x magnification. Direct spatial associations were then quantified using the quadrat counts from the registered whole slide images, allowing us to determine co-localization of cell types and/or cytokine expression. aITH was quantified using multi-region whole exome sequencing followed by TCR recognition potential predictions. The results from this analysis allowed us to test the model’s predictions about the timing and type of immune escape in colorectal cancer.

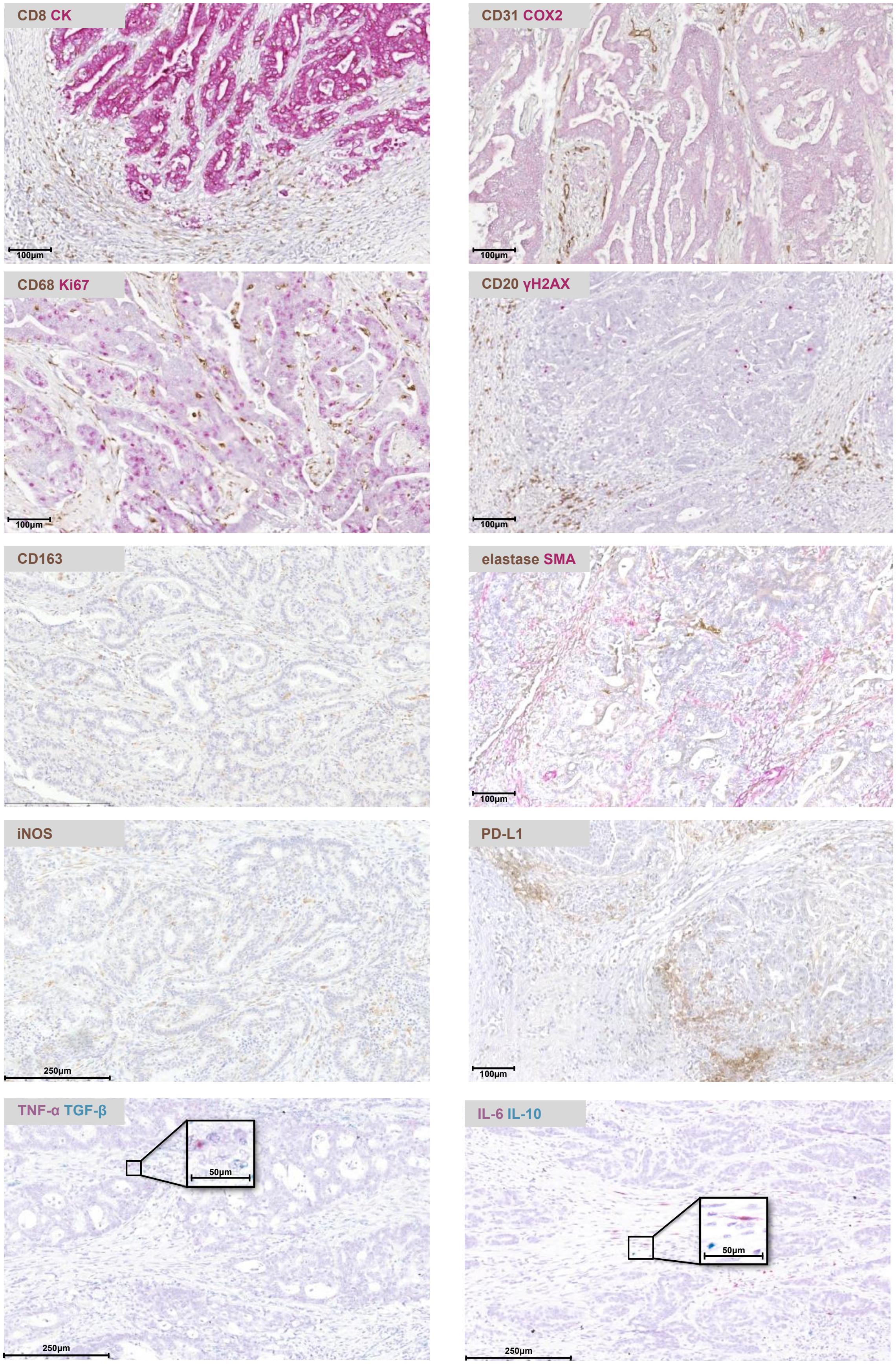

CD8 CK
100µm
CD31 COX2
100µm
CD68 Ki67
100µm
CD20 γH2AX
100µm
CD163
elastase SMA
100µm
iNOS
250µm
PD-L1
100µm
TNF-α TGF-β
50µm
250µm
IL-6 IL-10
50µm
250µm

Figure 3 The tumor immune ecology was described using 17 markers to identify tumor cells (CK), cytotoxic T-cells (CD8), macrophages (CD68), M2 macrophages (CD163), M1 macrophages (iNOS), neutrophils (elastase), B-cells (CD20), PD-L1, DNA damage (γH2AX), proliferation (Ki67), inflammatory cytokines (TNF-α, IL-6), and immunosuppressive cytokines (TGF-β, IL-10). These 10x images come from a CRC, but the analysis was conducted at 40x. CK, CD8, elastase, SMA, CD68, Ki67, CD20, γH2AX, CD31, COX2, and PD-L1 were stained for in n=12 colorectal adenomas (CRA), n=26, “carcinoma-in-adenoma” (CIA), and n=15 colorectal carcinomas (CRC). IL-6, IL-10, TNF-α, TGF-β, CD163, and iNOS were stained for in n=9 CRA, n=9 CIA, and n=9 CRC.

Computational image analysis

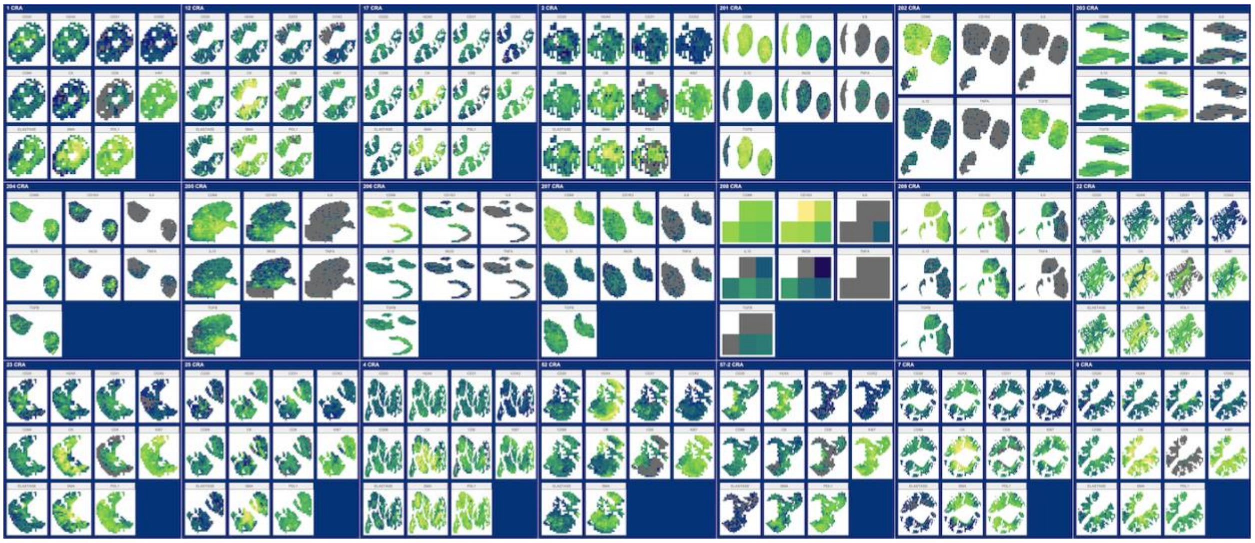

Figure 4 Quadrat counts for all CRA samples. Dark blues indicates low abundances, while yellow reflects higher abundances.

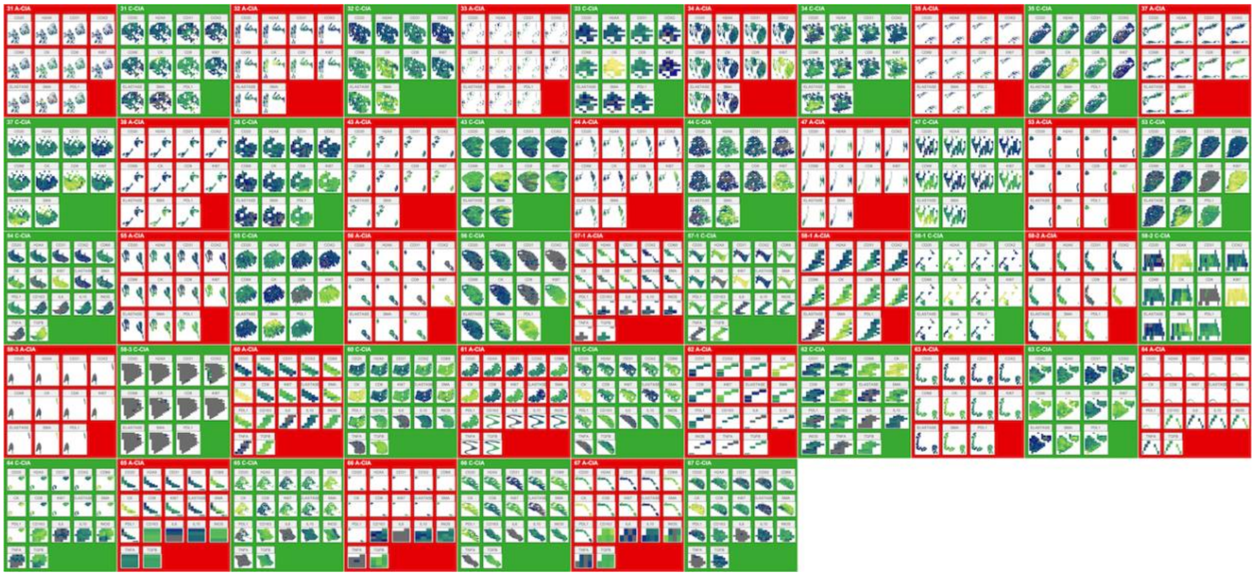

Figure 5. Quadrat counts for all CIA samples, with the CRA region in red, and the CRC region in green. Dark blues indicates low abundances, while yellow reflects higher abundances.

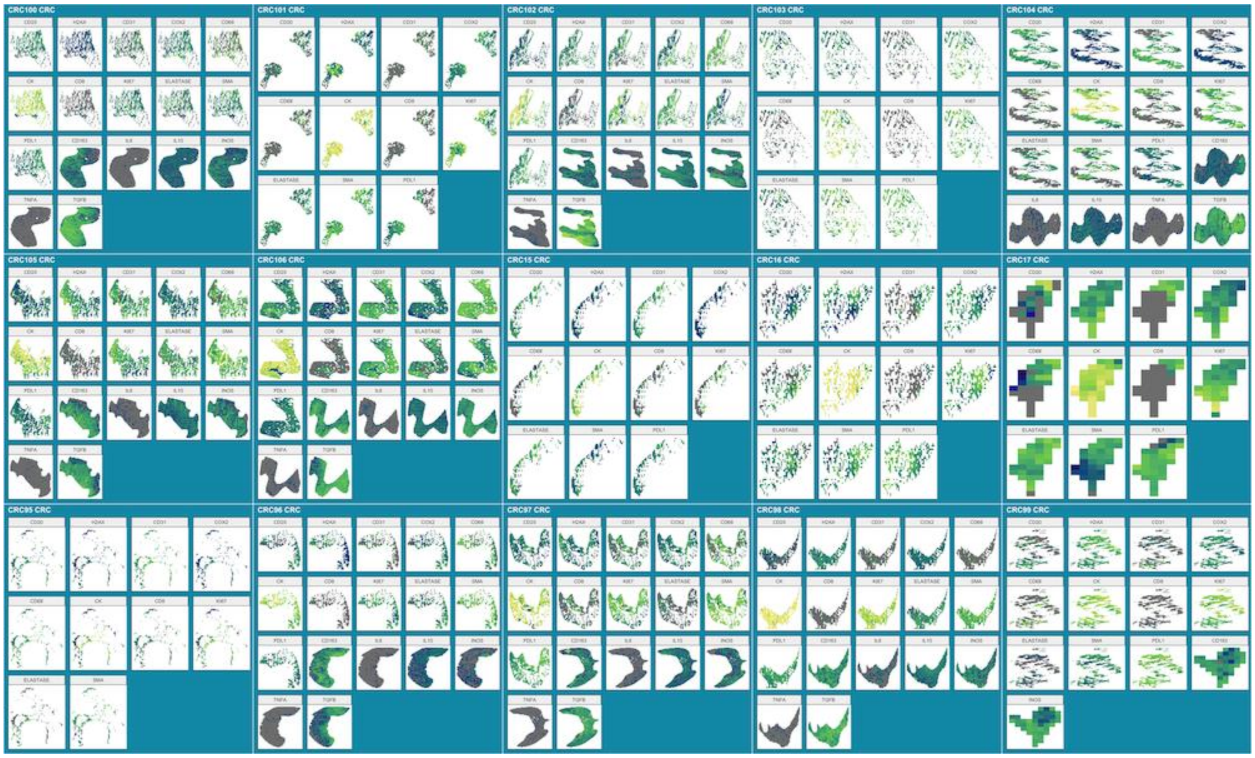

Figure 6. Quadrat counts for all CRC samples. Dark blues indicates low abundances, while yellow reflects higher abundances.

All image processing was conducted using OpenCV 4.1.2 (Bradski, 2000), scikit-image 0.18.3, (Walt et al., 2014), scikit-learn 0.23.1 (Pedregosa et al., 2011; Schreiber et al., 2011), and OpenSlide 3.4.1 (Goode, Gilbert, Harkes, Jukic, & Satyanarayanan, 2013) for the Python programming language. This research utilized Queen Mary's Apocrita HPC facility, supported by QMUL Research-IT (King, Butcher, & Zalewski, March, 2017).

Stain segmentation

The stains in each quadrat were separated using the following methods. In the first set of samples, three individuals (A.M.B, M.P.N., S.Y.H) conducted the staining, thus there was inter-batch variability and no single approach worked well for all. Thus, we trained a support vector machine (SVM) for each individual's collection of stained images. Four features were used to train and predict: the pixel's RGB values, and descriptor (SCD) (Khan, Rajpoot, Treanor, & Magee, 2014). In all cases, the accuracy of each SVM was above 90%. A final step to correct for misclassifications was to convert the each segmented image to HSV colorspace, and then reclassify brown pixels as red if their H >= 100, and red pixels as brown if 4 < H < 100. This algorithm used OpenCV (Bradski, 2000) colorspace conversion.

In the second sample set, all samples stained by the same person (A.M.B.), and the following method was used to separate red, brown, and green. The images were converted to cylindrical LAB colorspace (LCh) and saturation (S) calculated as the chroma normalized by lightness. All channels were then standardized to be in the range of 0-255. A pixel was considered green if all the following were true: h < 50 or h > 250, S >= 30. Likewise, a pixel was positive for red if 90 <= h < 145, S > 200. A pixel was classified as brown if 145 <= h < 210 and S >100. In this algorithm, scikit-image (Walt et al., 2014) was used to convert the RGB image to LAB.

Ecological Image Analysis

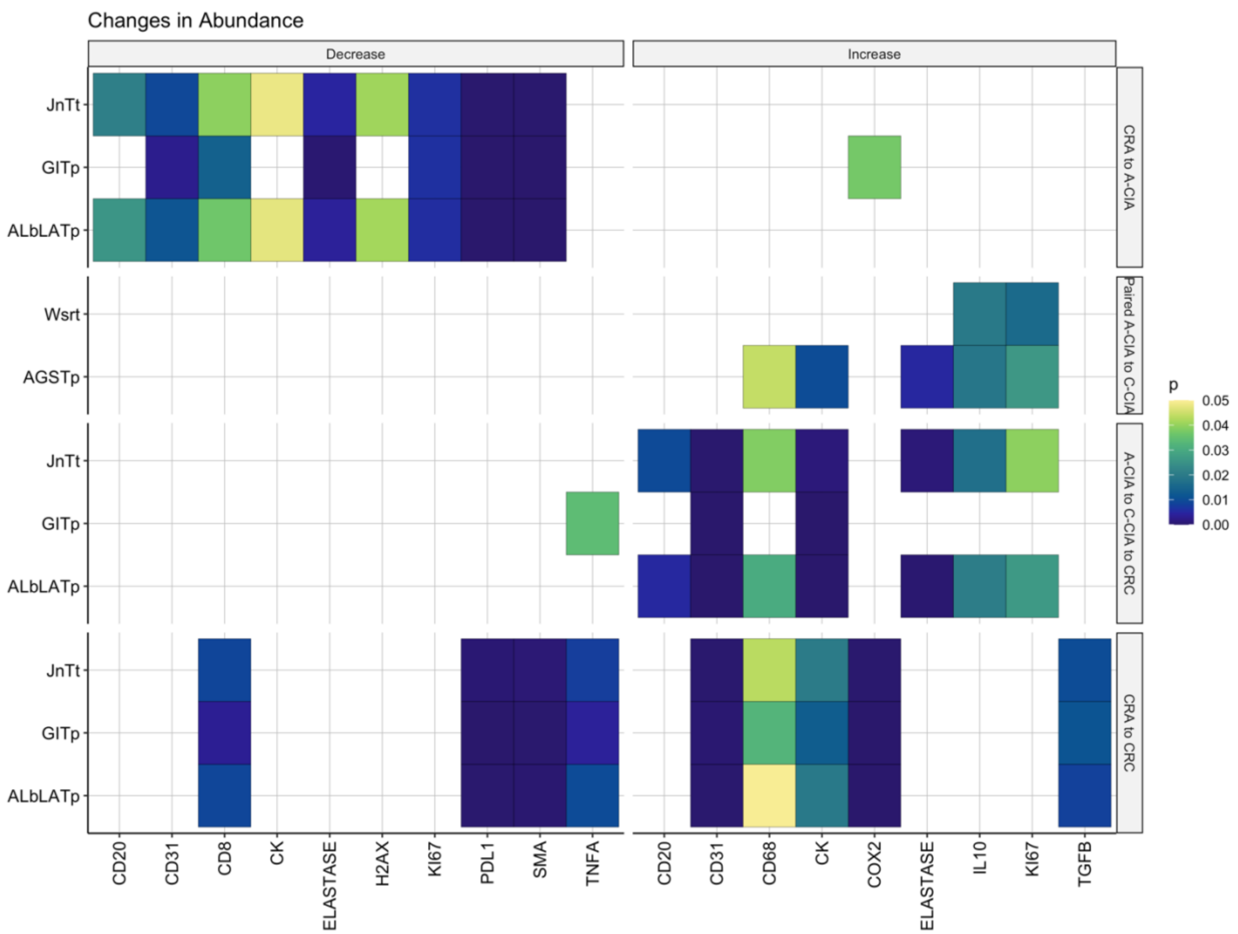

Figure 7. Significance tests for changes in abundance. A combination of frequentist statistics (y-axis) were used to determine if there were significant directional changes, where "Decrease" means there was a decrease in the abundance of a marker (x-axis) in each type of comparison. For example, all tests found a significant decrease in CD8 when comparing A-CIA to C-CIA to CRC. Paired tests were used to determine how abundances changed in the same tumor, from the A-CIA to its neighboring C-CIA. Colored tiles show significance, whereas missing tiles indicate non-significance. . KWrst= Kruskal-Wallis rank sum test, AKWTp=Approximative Kruskal-Wallis Test (permutation), GITp= General Independence Test (permutation), ALbLATp= Approximative Linear-by-Linear Association Test (permutation), JnTt= Jonckheere-Terpstra test.

Description of the tumor ecology within and across various stages was accomplished using a variety of methods. Cell abundances (assumed to be proportional to the number of pixels positive for each stain) were measured from the registered images, using the averaged quadrat counts (assumed to be proportional to the number of positive pixels) to determine if there were directional changes over time, i.e. if there were significant increases or decreases in abundance during progression from late adenoma to mature carcinoma. The significance of these trends was determined using a combination of frequentist and permutation statistical tests (Supplemental Figures 7 and 9). A similar analysis was conducted to determine if there were directional changes in spatial associations between the various cell types. Quadrat counts were used to construct a species association network (Popovic et al., 2019), whose coefficients were compared across stages. In addition to testing for these inter-stage trends, paired tests were used to determine if there were significant intra-tumor changes in cell abundance and interactions. This was accomplished by comparing the carcinoma (C-CIA) to its precursor adenoma (A-CIA) in the same CIA sample.

Several ecological tests were used to compare tumor-immune ecologies across stages. We quantified and compared the amount of ecological homogeneity within each tumor stage, using multivariate homogeneity of group dispersions (PERMDISP2)(M. J. Anderson, 2006). Permutational multivariate analysis of variance (PERMANOVA) was used to determine if there were significant differences in the structure of the ecological communities of each tumor stage, which was then visualized using constrained analysis of principal coordinates (CAP) (Marti J. Anderson & Willis, 2003; Oksanen et al., 2018). Using indicator

species analysis, we determined which, if any, cell types define the different tumor stages (De Cáceres & Legendre, 2009). Finally, the Mantel test was used to determine if differences in the immune ecology and microenvironment are correlated (Legendre & Legendre, 2012), as might be expected to occur during tumor instigated immune remodeling.

A distance matrix, which compares tumors based on the whole collection of markers, is required for many of the ecological tests we performed. We tested all combinations of distance metrics and normalization methods in the *vegan* R package (version 2.5-7) (Oksanen et al., 2018) and found that the Jaccard distance on log-scaled PPC most often had adjacent adenomas in CIA samples as being the most similar to one another, as would be expected. It should be noted that the Jaccard distance in the *vegan* package is not the same as a traditional Jaccard distance, but more of a variant of the Bray-Curtis distance. Thus, we performed the analysis on log-scaled data and a distance matrix constructed using the Jaccard distance. PERMANOVA, PERMDSIP2, CAP, and the Mantel tests were all conducted on this distance matrix.

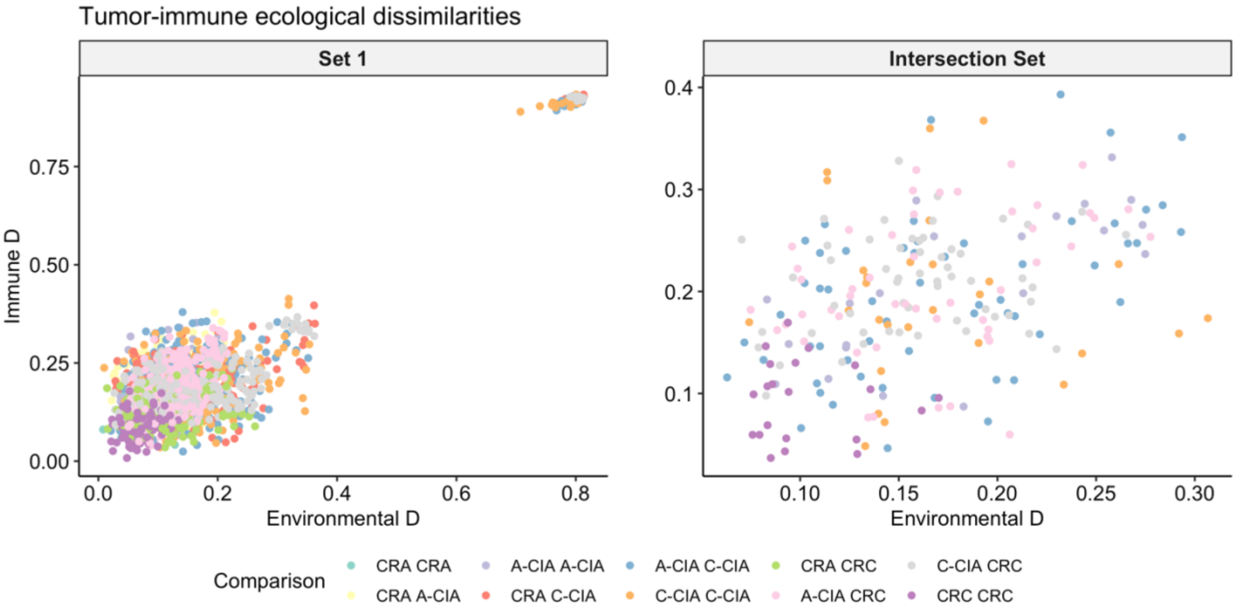

Figure 8 Differences in immune composition (cytotoxic T-cells, B-cells, macrophages, and neutrophils) and environment (tumor cells, vasculature, PDL1, and fibroblasts) are significantly correlated, as determined by the Mantel test. This indicates the immune system and tumor microenvironment change together, consistent with a scenario in which tumors create an immunosuppressive niche that further promotes tumor growth.

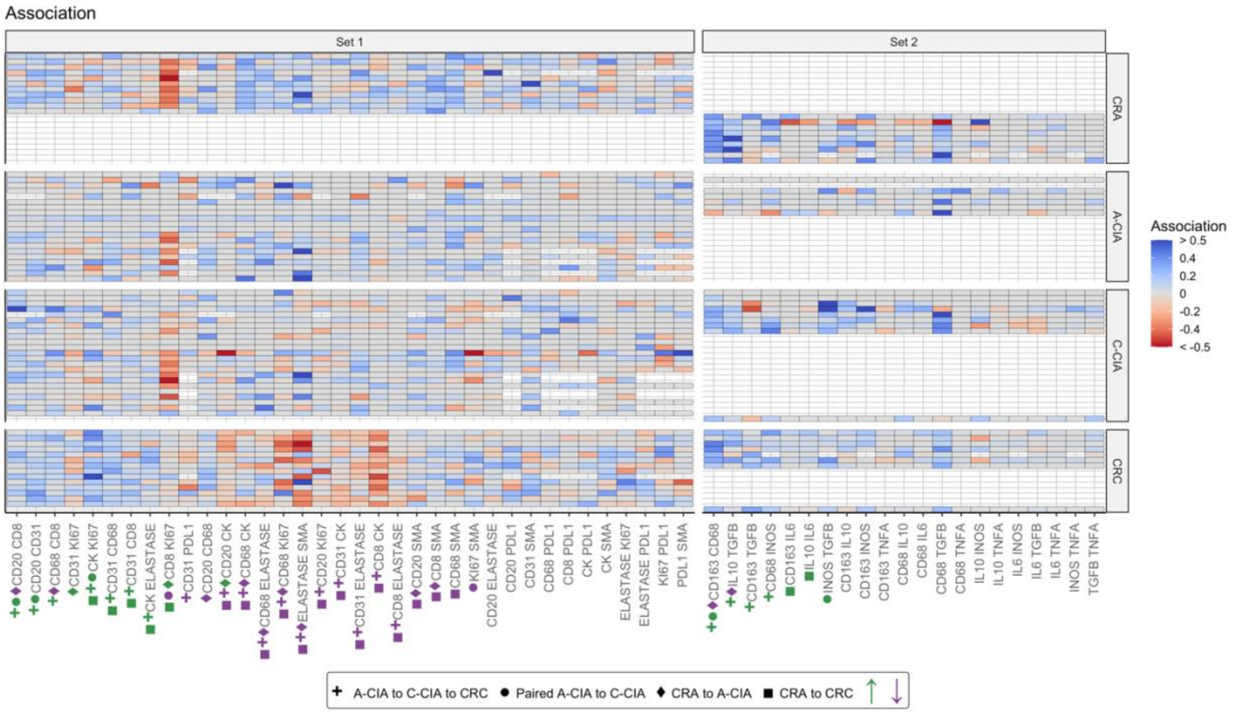

Figure 9. Table showing significant changes in direct pairwise cell-cell/cell-cytokine spatial associations, where each row is a sample, and each column a spatial association. Shapes below each marker pair signify that there were significant changes in spatial association across the specified group. Green indicates that the association increased from the first group to the last group, while purple indicates that the spatial association decreased. Here, surprisingly, we observed that, compared to A-CIA, CRA have a higher association of CD68/CD163 (purple diamond), indicative of M2 macrophages, and a greater association between TGF-β & IL-10, suggesting CRA are more immunosuppressive than A-CIA. Interestingly, the same trend of increased CD68/CD163 and IL-10/ TGF-β are observed during the evolution of the tumor from progressed adenoma to carcinoma. A notable difference is that CD8 more strongly spatially co-localized with tumor (CK) in CRA compared to A-CIA and CRC (purple diamond and square), and the association between CD8 and tumor decreases from progressed adenoma to CRC (purple plus). This suggests that while CRA may have some immunosuppressive cells and cytokines, they remain most immunogenic.

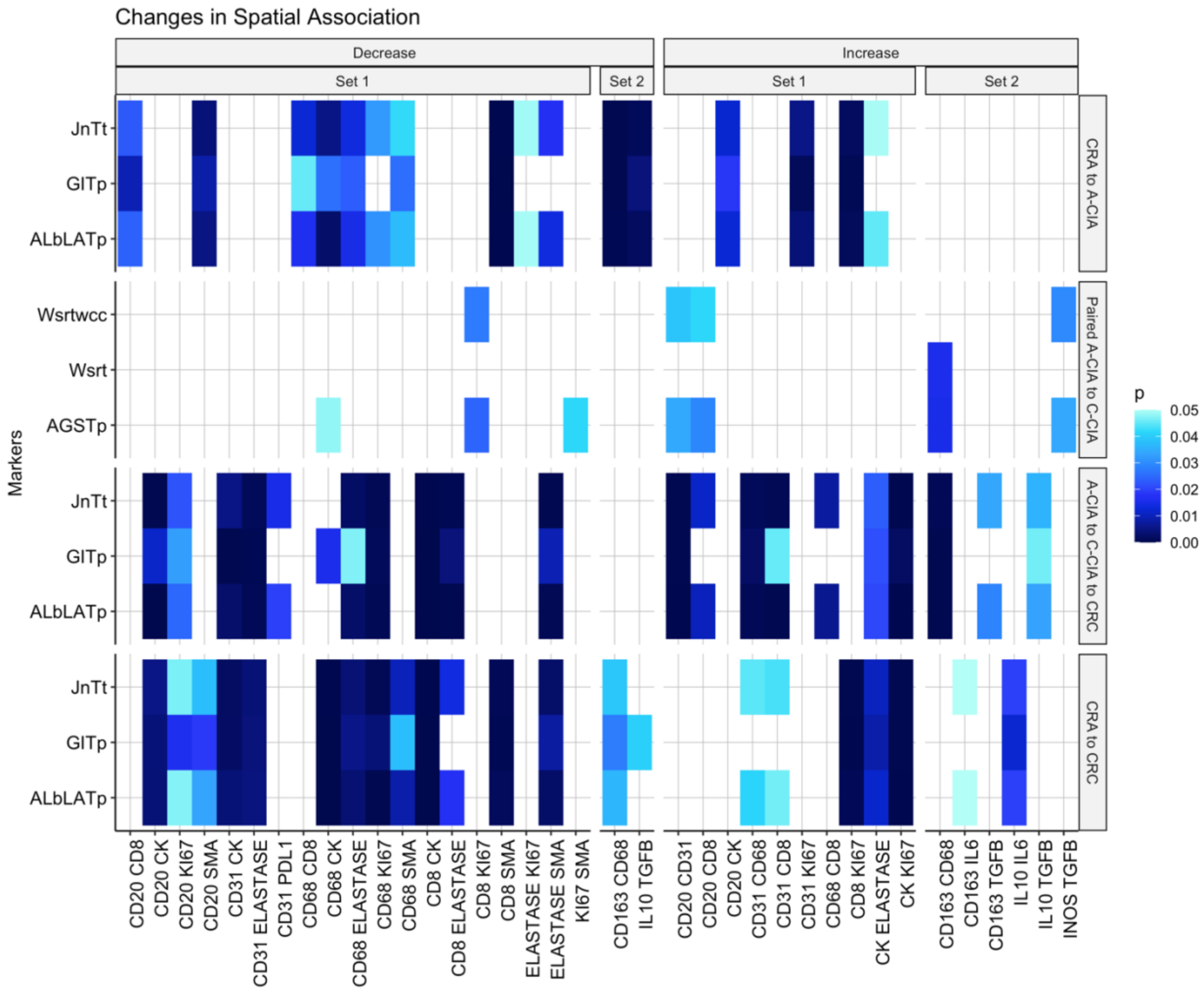

Figure S10. Significance tests for changes in spatial associations. A combination of frequentist statistics (y-axis) were used to determine if there were significant directional changes, where "Decrease" means there was a decrease in the abundance of a marker (x-axis) in each type of comparison. For example, all tests found a significant decrease in the spatial association between CD8 and CK when comparing A-CIA to C-CIA to CRC. Paired tests were used to determine how abundances changed in the same tumor, from the A-CIA to its neighboring C-CIA. Colored tiles show significance, whereas missing tiles indicate non-significance. KWrst= Kruskal-Wallis rank sum test, AKWTp=Approximative Kruskal-Wallis Test (permutation), GITp= General Independence Test (permutation), ALbLATp= Approximative Linear-by-Linear Association Test (permutation), JnTt= Jonckheere-Terpstra test.

A suite of statistical tests was used to detect significant changes in marker positivity, co-localization, and direct cell-cell interactions (Supplemental Figure 7 and 9). Several methods were used to find those results that were robust, as those that were found to be significant across multiple tests are most likely to be true. The two-sided Kruskal-Wallis rank sum test, two-sided approximate Kruskal-Wallis Test, and two-sided General Independence Test were used to determine there were differences between the tumor subtypes CRA, A-CIA, C-CIA, and CRC. Assuming that the carcinomas will take over the adenomas in the ca-in-ad samples, and thus develop into carcinomas, one can test for trends in the data by setting the timing order of A-CIA-> C-CIA -> CRC. Having ordered the subtypes, one-sided Approximative Linear-by-Linear Association Test and the Jonckheere-Terpstra test were used to determine if there a significant increase or decrease across groups (Seshan, 2018). The Conover-Iman test of multiple comparisons using rank sums was used as the post-hoc test to determine which subtypes were significantly different from one another (Dinno, 2017).

The paired-tests looking for differences between A-CIA and C-CIA included the General Symmetry test and the Wilcoxon rank sum test. As with the tests comparing all subtypes, the two-sided version of these tests was used to detect differences, and the one-sided test was used to detect the presence of trends and the direction of those trends.

The two-sided approximate Kruskal-Wallis Test, General Independence Test, General Symmetry test, and Approximative Linear-by-Linear Association Tests are permutation tests and were conducted using *coin* package for R (version 1.4-2) (Hothorn, Hornik, Wiel, & Zeileis, 2008).

Model Fitting

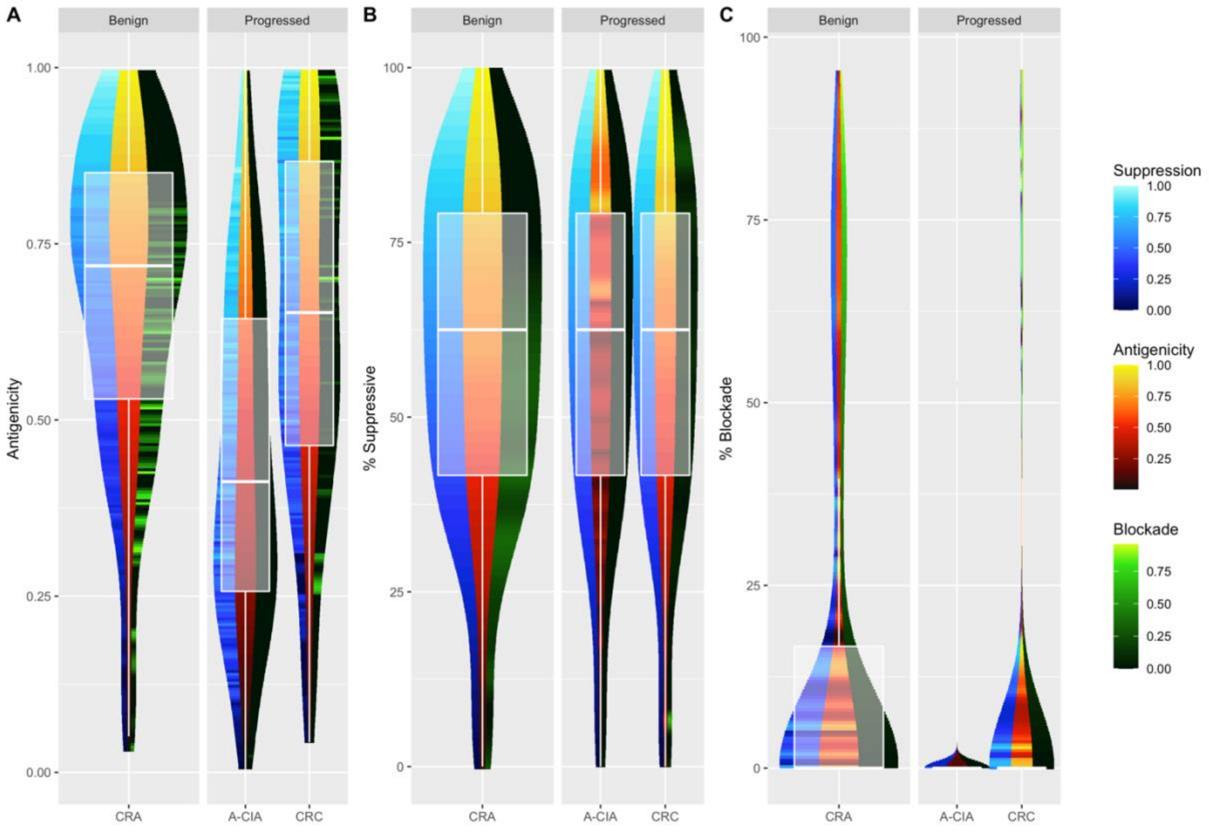

Figure 11 Distribution of antigenicities (A), and percent of the tumor with immunosuppressive cell infiltrate (B), and percent of the tumor with blockade (PD-L1) (C), in benign adenomas compared to those of progressed adenomas and carcinomas using the subset of parameters that matched the data. The horizontal bin in each violin is colored by the average value of phenotypes that are within that bin. For each parameter combination that reproduced the observed patterns, the two most different malignant and benign tumors were selected for plotting.

Supplementary table 1 – Sample details

Sample number	Classification	Site	Used for WES	Used for IHC
**1**	Adenoma	Oxford		Y
**2**	Adenoma	Oxford		Y
**4**	Adenoma	Oxford	Y	Y
**6**	Adenoma	Oxford	Y	
**7**	Adenoma	Oxford		Y
**8**	Adenoma	Oxford		Y
**12**	Adenoma	Oxford	Y	Y
**17**	Adenoma	Oxford		Y
**22**	Adenoma	Oxford	Y	Y
**23**	Adenoma	Oxford		Y
**25**	Adenoma	Oxford		Y
**52**	Adenoma	UCH		Y
**57-2**	Adenoma	UCH		Y
**K**	Adenoma		Y	
**S**	Adenoma		Y	
**P**	Adenoma		Y	
**31**	Ca-in-ad	Oxford		Y
**32**	Ca-in-ad	Oxford	Y	Y
**33**	Ca-in-ad	Oxford	Y	Y
**34**	Ca-in-ad	Oxford		Y
**35**	Ca-in-ad	Oxford		Y
**37**	Ca-in-ad	Oxford		Y

**38**	Ca-in-ad	Oxford		Y
**43**	Ca-in-ad	Oxford		Y
**44**	Ca-in-ad	Oxford		Y
**47**	Ca-in-ad	Oxford	Y	Y
**53**	Ca-in-ad	UCH		Y
**55**	Ca-in-ad	UCH		Y
**56**	Ca-in-ad	UCH		Y
**57-1**	Ca-in-ad	UCH		Y
**58-2**	Ca-in-ad	UCH		Y
**58-3**	Ca-in-ad	UCH		Y
**60**	Ca-in-ad	UCH		Y
**61**	Ca-in-ad	UCH		Y
**62**	Ca-in-ad	UCH		Y
**63**	Ca-in-ad	UCH		Y
**64**	Ca-in-ad	UCH		Y
**65**	Ca-in-ad	UCH		Y
**66**	Ca-in-ad	UCH		Y
**67**	Ca-in-ad	UCH		Y
**15**	Carcinoma	Oxford	Y	Y
**16**	Carcinoma	Oxford	Y	Y
**17**	Carcinoma	Oxford	Y	Y
**54**	Carcinoma	UCH		Y
**58-1**	Carcinoma	UCH		Y
**95**	Carcinoma	UCH		Y
**96**	Carcinoma	UCH		Y
**97**	Carcinoma	UCH		Y
**98**	Carcinoma	UCH		Y

**99**	Carcinoma	UCH		Y
**100**	Carcinoma	UCH		Y
**101**	Carcinoma	UCH		Y
**102**	Carcinoma	UCH		Y
**103**	Carcinoma	UCH		Y
**104**	Carcinoma	UCH		Y
**105**	Carcinoma	UCH		Y
**106**	Carcinoma	UCH		Y
**M**	Carcinoma		Y	
**N**	Carcinoma		Y	
**T**	Carcinoma		Y	
**G**	Carcinoma		Y	
**W**	Carcinoma		Y	

Supplementary table 2 – Antibody details

Slide no. (antibody no.)	Antibody	Manufacturer (cat no.)	Dilution	Antigen Retrieval
**1 (1)**	Monoclonal mouse anti-human CD8	Dako (M7103)	1:100<br><br>1hr RT	Sodium citrate, pH 6.0

**1 (2)**	Monoclonal mouse anti-human cytokeratin	Dako (M3515)	1:100<br>1hr RT	Sodium citrate, pH 6.0
**2 (1)**	Monoclonal mouse anti-human CD20	Dako (M0755)	1:300<br>1hr RT	Sodium citrate, pH 6.0
**2 (2)**	Monoclonal mouse anti-human γH2AX	Abcam (ab26350)	1:500<br>1hr RT	Sodium citrate, pH 6.0
**3 (1)**	Monoclonal mouse anti-human CD68	Dako (M0876)	1:100<br>1hr RT	Sodium citrate, pH 6.0
**3 (2)**	Monoclonal rabbit anti-human Ki67	Abcam (ab16667)	1:2000<br>1hr RT	Sodium citrate, pH 6.0
**4 (1)**	Monoclonal mouse anti-human neutrophil elastase	Dako (M0752)	1:20<br>1hr RT	None
**4 (2)**	Monoclonal mouse anti-α-smooth muscle actin (SMA)	Sigma (A2547)	1:3000<br>1hr RT	Sodium citrate, pH 6.0
**5 (1)**	Monoclonal mouse anti-human CD31	Novocastra (CD31-1A10)	1:100<br>O/N 4ºC	Sodium citrate, pH 6.0
**6 (1)**	Monoclonal rabbit anti-human PD-L1	Cell Signaling Technology (E1L3N)	1:400<br>1hr RT	Tris-EDTA, pH 9.0
**Set 2**	Polyclonal rabbit anti-iNOS	Abcam (ab15323)	1:100<br>1hr RT	Tris-EDTA, pH 9.0
**Set 2**	Monoclonal mouse anti-CD163	Novus (NB110-59935)	1:100<br>1hr RT	Tris-EDTA, pH 9.0

**-**	Polyclonal goat anti-rabbit IgG biotinylated	Dako (E0432)	1:400<br><br>45min RT	-
**-**	Polyclonal rabbit anti-mouse IgG biotinylated	Dako (E0354)	1:400<br><br>45min RT	-
**-**	Streptavidin-HRP	Dako (P0397)	1:500<br><br>45min RT	-
**-**	Streptavidin-AP	Life Technologies (S921)	1:500<br><br>45min RT	-
