# Supplementary material for: Immunosuppressive niche engineering at the onset of human colorectal cancer
Source: Nat Commun. 2022 Apr 4;13:1798. doi: 10.1038/s41467-022-29027-8 (PMC8979971; doi:10.1038/s41467-022-29027-8)
Supplement: Supplementary file 2 — Reporting Summary [file 41467_2022_29027_MOESM2_ESM.pdf]

naturereseach

Corresponding author(s): Dr. Alexander Anderson

Last updated by author(s): Feb 16, 2022

Reporting Summary

Nature Research wishes to improve the reproducibility of the work that we publish. This form provides structure for consistency and transparency in reporting. For further information on Nature Research policies, seeAuthors & Referees and theEditorial Policy Checklist .

Statistics

For all statistical analyses, confirm that the following items are present in the figure legend, table legend, main text, or Methods section.

n/a	Confirmed	
☐	☒	The exact sample size (*n*) for each experimental group/condition, given as a discrete number and unit of measurement
☐	☒	A statement on whether measurements were taken from distinct samples or whether the same sample was measured repeatedly
☐	☒	The statistical test(s) used AND whether they are one- or two-sided<br>*Only common tests should be described solely by name; describe more complex techniques in the Methods section.*
☒	☐	A description of all covariates tested
☐	☒	A description of any assumptions or corrections, such as tests of normality and adjustment for multiple comparisons
☐	☒	A full description of the statistical parameters including central tendency (e.g. means) or other basic estimates (e.g. regression coefficient) AND variation (e.g. standard deviation) or associated estimates of uncertainty (e.g. confidence intervals)
☐	☒	For null hypothesis testing, the test statistic (e.g. *F*, *t*, *r*) with confidence intervals, effect sizes, degrees of freedom and *P* value noted<br>*Give P values as exact values whenever suitable.*
☒	☐	For Bayesian analysis, information on the choice of priors and Markov chain Monte Carlo settings
☒	☐	For hierarchical and complex designs, identification of the appropriate level for tests and full reporting of outcomes
☒	☐	Estimates of effect sizes (e.g. Cohen's *d*, Pearson's *r*), indicating how they were calculated

Our web collection on statistics for biologists contains articles on many of the points above.

Software and code

Policy information about availability of computer code

Data collection	No software was used to collect data.
Data analysis	Data analysis and simulations used open source packages available for R 3.6.0 and Python 3.The custom code used to conduct simulations has been made available on GitHub (https://github.com/MathOnco/gatenbee_2022_immunosuppressive_niche) and assigned a DOI using Zenodo (10.5281/zenodo.5974873).<br><br>R packages: vegan (2.5-7), coin (1.4-2), ecoCopula (1.0.1)<br>Python packages: OpenCV (4.1.2), scikit-image (0.18.3), scikit-learn (0.23.1), OpenSlide (3.4.1), NeoPredPipe (1.0), leidenalg (0.8.2)<br>C: samtools 1.2<br>C++: multisnv 2<br>Other: NetMHCpan (4.0), PolySolver, PICARD (2.17)

For manuscripts utilizing custom algorithms or software that are central to the research but not yet described in published literature, software must be made available to editors/reviewers. We strongly encourage code deposition in a community repository (e.g. GitHub). See the Nature Research guidelines for submitting code & software for further information.

Data

Policy information about availability of data

All manuscripts must include a data availability statement. This statement should provide the following information, where applicable:
- Accession codes, unique identifiers, or web links for publicly available datasets
- A list of figures that have associated raw data
- A description of any restrictions on data availability

The raw data used to conduct all statistical tests has been made available on Zenodo (https://zenodo.org/record/5974913#.YgFiT1jMLyw). This data was used to create figures 4-9.

Field-specific reporting

Please select the one below that is the best fit for your research. If you are not sure, read the appropriate sections before making your selection.

☒ Life sciences ☐ Behavioural & social sciences ☐ Ecological, evolutionary & environmental sciences

For a reference copy of the document with all sections, see nature.com/documents/nr-reporting-summary-flat.pdf

Life sciences study design

All studies must disclose on these points even when the disclosure is negative.

Sample size	The number of samples used was based on availability
Data exclusions	All samples were included
Replication	N/A. No experiments were performed. Analysis was based on resected tumors
Randomization	N/A. The study was observational. No interventions were performed and so randomization was not required
Blinding	N/A. Tumor stages were compared, requiring that they be known for analysis.

Reporting for specific materials, systems and methods

We require information from authors about some types of materials, experimental systems and methods used in many studies. Here, indicate whether each material, system or method listed is relevant to your study. If you are not sure if a list item applies to your research, read the appropriate section before selecting a response.

Materials & experimental systems

n/a	Involved in the study	
☐	☒	Antibodies
☒	☐	Eukaryotic cell lines
☒	☐	Palaeontology
☒	☐	Animals and other organisms
☐	☒	Human research participants
☒	☐	Clinical data

Methods

n/a	Involved in the study	
☒	☐	ChIP-seq
☒	☐	Flow cytometry
☒	☐	MRI-based neuroimaging

Antibodies

Antibodies used

Monoclonal mouse anti-human CD8, Dako (M7103)
Monoclonal mouse anti-human cytokeratin, Dako (M3515)
Monoclonal mouse anti-human CD20, Dako (M0755)
Monoclonal mouse anti-human γH2AX, Abcam (ab26350)
Monoclonal mouse anti-human CD68, Dako (M0876)
Monoclonal rabbit anti-human Ki67, Abcam (ab16667)
Monoclonal mouse anti-human neutrophil elastase, Dako (M0752)
Monoclonal mouse anti-α-smooth muscle actin (SMA), Sigma (A2547)
Monoclonal mouse anti-human CD31, Novocastra (CD31-1A10)
Monoclonal rabbit anti-human PD-L1, Cell Signaling Technology (E1L3N)
Polyclonal goat anti-rabbit IgG biotinylated, Dako (E0432)
Polyclonal rabbit anti-mouse IgG biotinylated, Dako (E0354)
Polyclonal rabbit anti-iNOS, Abcam (ab15323)
Monoclonal mouse anti-CD163, Novus (NB110-59935)
Polyclonal goat anti-rabbit IgG biotinylated, Dako (E0432)
Polyclonal rabbit anti-mouse IgG biotinylated, Dako (E0354)
Streptavidin-HRP, Dako (P0397)
Streptavidin-AP, Life Technologies (S921)

Validation	All antibodies were validated by the manufacturers for immunohistochemistry on human FFPE samples. Furthermore, we validated our protocols and antibody dilutions in-house (see Table S2) using positive control tissue of human FFPE tonsil or human FFPE carcinoma where appropriate.

Human research participants

Policy information about studies involving human research participants

Population characteristics	N/A. The study was performed on anonymized archival specimens
Recruitment	N/A. Samples were collected from a pathology archive
Ethics oversight	Samples were either collected at University College Hospital, London, under UK ethical approval (07/Q1604/17), or John Radcliffe Hospital, Oxford under ethical approval (10/H0604/72). Written informed consent was waived by the relevant RECs due to the retrospective and anonymous nature of this study.

Note that full information on the approval of the study protocol must also be provided in the manuscript.
